# LncRNA RPSAP52 promotes cell proliferation and inhibits cell apoptosis via modulating miR-665/STAT3 in gastric cancer

**DOI:** 10.1080/21655979.2022.2054754

**Published:** 2022-03-24

**Authors:** Chao He, Yuanyuan Liu, Jinhou Li, Xiao Zheng, Jianwei Liang, Gang Cui, Hong Chang

**Affiliations:** a Department of Hepatobiliary Surgery, Shandong Provincial Hospital, Cheeloo College of Medicine, Shandong University, Jinan China; b Department of Gastrointestinal Surgery, Taian Central Hospital, Taian China; c Department of Respiratory Medicine, The Second Affiliated Hospital of Shandong First Medical University, Taian China; d Department of Hepatobiliary and Pancreatic, Taian Central Hospital, Taian China

**Keywords:** LncRNA RPSAP52, gastric cancer, miR-665, STAT3, cell proliferation, cell apoptosis

## Abstract

LncRNA RPSAP52 is a newly identified functional molecular in several cancers, but its role in gastric cancer (GC) is currently unclear. This study aimed to investigate the biofunction of lncRNA RPSAP52 in GC. Quantitative polymerase-chain reaction (RT-qPCR) was employed to analyze the gene level of lncRNA RPSAP52 and miR-665. Cell proliferation capacity was evaluated via CCK-8 and colony formation assay. Flow cytometry was applied to detect cell cycle and cell apoptosis. Hematoxylin-eosin staining was conducted for histopathological analysis. Immunochemical staining was carried out to detect expression level of ki-67. Subcellular fractionation was performed to explore the position of lncRNA RPSAP52. The binding relationship among lncRNA RPSAP52, miR-665 and STAT3 was verified via luciferase reporter assay. RNA pull down experiments were used to verify the binding relationship between lncRNA RPSAP52 and miR-665. The STAT3 level was evaluated via Western blot. LncRNA RPSAP52 is significantly elevated in GC cells. Deletion of lncRNA RPSAP52 restrained cell proliferation and induced G0-G1 phase arrest, while expediting apoptosis in GC cells. Tumor growth in vivo was suppressed following lncRNA RPSAP52 depletion. MiR-665 was verified as the target of lncRNA RPSAP52. A ceRNA-sponge mechanism of lncRNA RPSAP52 on miR-665 was identified. Meanwhile, miR-665 functions as STAT3 sponge. MiR-665 overexpression and STAT3 depletion served the same functions as lncRNA RPSAP52 depletion in GC cells. LncRNA RPSAP52 exerted anti-cancer effects via modulating miR-665/STAT3 in GC.

**Abbreviations**: Gastric cancer (GC); Quantitative polymerase-chain reaction (RT-qPCR); Helicobacter pylori (H. pylori); Roswell Park Memorial Institute 1640 (RPMI 1640); fetal bovine serum (FBS); glyceraldheyde 3-phosphate dehydrogenase (GAPDH); propidium iodide (PI); Cell counting kit-8 (CCK-8); radioimmunoprecipitation assay (RIPA); sodium dodecyl sulfate-polyacrylamide gel electrophoresis (SDS-PAGE); polyvinylidene fluoride (PVDF); enhanced chemiluminescence (ECL); Statistical Product and Service Solutions (SPSS); standard deviation (SD).

## Graphical abstract

**Figure:**
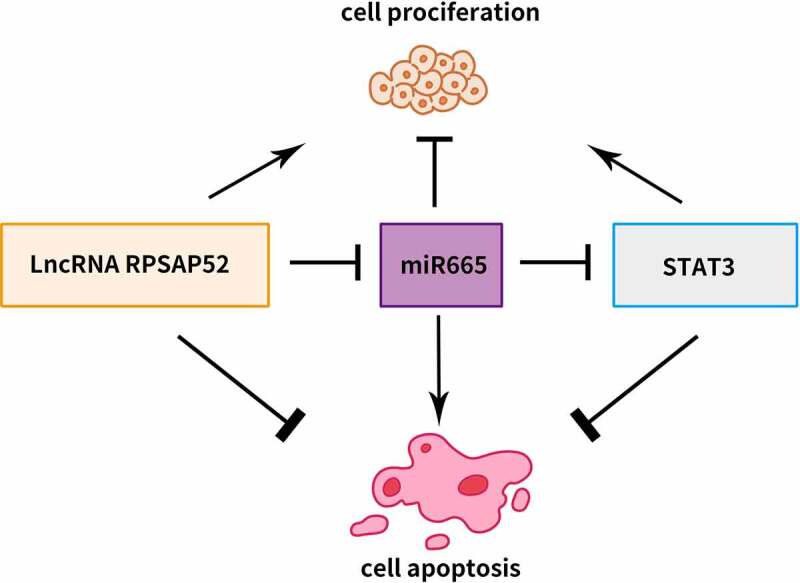

## Introduction

Gastric cancer (GC) is one of the world’s most lethal and common malignancies with a high incidence in China, placing a crushing economic and healthy burden on people [1,2]. The pathogenic factors of GC are quite complicated, including diet, environment, heredity, Helicobacter pylori (H. pylori) infection and so forth [^3–6^]. The early clinical manifestations of GC are atypical and vague, making it extremely difficult for early diagnosis. The five-year overall survival rate of patients with advanced metastasis GC is poor, whereas, early-stage localized GC patients has a 5-year overall survival rate of more than 50% [7]. Accordingly, development of new effective therapeutic targets and diagnostic markers is pressing.

LncRNA has been once considered as the transcriptional garbage without biological function. But an accumulating body of research indicates that lncRNA functions as a crucial participant in cell proliferation, invasion, migration, metastasis and so forth in various type of cancer [^8–12^]. LncRNA has been confirmed to work by regulating chromatin organization, gene transcription, gene expression and gene splicing in cancer [9,13,14]. lncRNA has been demonstrated as a rising star in GC. Numerous lncRNAs have been identified as the promising candidates for diagnosis or therapeutic target in GC, such as LncRNA CTD-2510F5.4, lncRNA MAGI2-AS3, lncRNA HOXC-AS3, lncRNA MYOSLID and LINC01485 [^15–19^].

LncRNA RPSAP52 has been confirmed as an oncogene in glioblastoma [20]. LncRNA RPSAP52 is also identified as a novel player in pancreatic cancer [21]. Additionally, lncRNA RPSAP52 is involved in pituitary tumorigenesis [22]. Nevertheless, the impacts of lncRNA RPSAP52 in GC remain uncharacterized and has been first examined in this report.

MiRNAs, another pivotal regulator in cancer, always acts as the downstream target of lncRNA. As predicted by bioinformatics tools, miRNA-665 is a target of lncRNA RPSAP52 and could target STAT3. MiRNA-665 is a novel regulator involved in the epithelial–mesenchymal transition of GC [23].

Given the important role of lncRNA RPSAP52 in multiple cancers, we hypothesized that it also promotes the oncogenic function of gastric cancer cells. We hypothesized that the lncRNA RPSAP52 could sponge up miR-665, thereby possibly regulating STAT3. This study aimed to investigate the mechanism of lncRNA RPSAP52-mediated miR-665/STAT3 signaling pathway in gastric cancer, and to propose new targets for the treatment of gastric cancer.

## Methods and materials

### Cell culture and transfection

The GES-1, MKN-28, SGC-7901, AGS, and BGC-823 cells were procured form Procell Life Science&Technology Co., Ltd (Wuhan, China). All the cells (1 × 10^4^) were cultured in Roswell Park Memorial Institute 1640 (RPMI 1640) medium supplemented with penicillin-streptomycin (1%) and fetal bovine serum (FBS) (10%), at 37°C in an incubator with 5% CO_2_. Replacement of culture substrate was performed every other day. The transfection was performed when the cells reached 90% confluency. The cells were transfected with lncRNA RPSAP52-shRNA#1, lncRNA RPSAP52-shRNA#2, miR-665 mimics, STAT3-shRNA#1, STAT3-shRNA#2, respectively, via using Lipofectamine 3000 (Invitrogen, Carlsbad, CA, USA) according to the guidance of manufacturer.

### Colony formation assay

The cells of logarithmic phase in study groups were all digested with 0.25% trypsin. Then, the cells were dissociated with plastic pipette and resuspended in the RPMI 1640 medium with 10% FBS. The cells were seeded in the six-well plate at a density of 1 × 10^3^ in the incubator for 1 week. Subsequently, the cells were fixed with paraformaldehyde (4%) for 15 min and stained with crystalline violet (0.1%) for half an hour. The colony formation capacity was assessed via using a microscope

### RT-qPCR assay

The cells after treatment in the study group were collected and the total RNAs were isolated via using RNAprep pure Cell Kit (TIANGEN Biotech Co., Ltd., Beijing, China). The reverse transcription was conducted via using PrimeScript™ RT-PCR Kit (TaKaRa, Tokyo, Japan) in accordance with the guidance of manufacturer. The PCR assay was processed on a StepOne Real-time PCR System (Applied Biosystems, Foster City, CA, USA). U6 and glyceraldheyde 3-phosphate dehydrogenase (GAPDH) were used as the internal reference. The primers were listed as follows: RPSAP52: 5-'GAGCAAACACATCGGAGACA-3' (Forward), 5-'AATTGGATTCCCACTGCAAG-3' (Reverse); miR-665: 5'-GGTGAACCAGGAGGCTGAGG-3' (Forward), 5'-CAGTGCAGGGTCCGAGGTAT-3' (Reverse). The gene expression level was assessed via 2− ΔΔCt method.

### Flow cytometry assay

The assessment of cell apoptosis and cell cycle were conducted via flow cytometry assay. For assessment of cell apoptosis, the cells after transfection in the study groups were collected, washed, and resuspended in the binding buffer (400 μL). Then, 10 µL Annexin V-FITC and 10 µL propidium iodide (PI) were incubated with the cells for 15 min away from light. For cell cycle analysis, the cells after transfection were washed with PBS. After centrifugation and resuspension, the cells were submitted to fixation with 75% ethanol overnight. Subsequently, after rinse with PBS and centrifugation, the cells were incubated with 100 µL RNase (Sigma, St. Louis, MO, USA) for half an hour, at 37°C. Then, 400 µL PI was added to stain the cells for half an hour in the dark. The results of cell apoptosis and cell cycle were analyzed on flow cytometry (FACS Calibur, BD, Franklin Lakes, NJ, USA).

### Cell counting kit-8 (CCK-8) assay

The CCK-8 assay was conducted for assessment of cell proliferation. After transfection for 48 h, the cells (1 × 10^4^) were seeded into the 96-well plate and were incubated for 24 h and 48 h. Then, CCK-8 solution (Sigma, St. Louis, MO, USA, 10 μL) was added and incubated with the cells for 2 h at 37°C. The absorbance at a wave length 490 nm was measured in a microplate instrument (Bio-Rad, USA).

### Subcellular fractionation

The location of lncRNA RPSAP52 was determined via subcellular fractionation assay using cytoplasmic and nuclear RNA Purification Kit (Cat. 21000, 37400, Norgen Biotek, Thorold, ON, Canada) in accordance with the guidance of manufacturer. U6 worked as nucleus control and GAPDH as cytoplasm control. The lncRNA RPSAP52 in the fractions of cytoplasmic and nucleus was detected via qRT-PCR assay.

### Luciferase reporter assay

The validation of the target was performed via luciferase reporter assay. The mutant type of lncRNA RPSAP52/STAT3 harboring the binding site was generated using site-directed mutagenesis kit (Beyotime, Shanghai, China). The reporter plasmid was constructed via cloning the wild type (WT) lncRNA RPSAP52/STAT3 or mutant type (MUT) RPSAP52/STAT3 binding sequence into pmirGLO vector (Promega, Madison, WI, USA), respectively. The WT/MUT lncRNA RPSAP52 or WT/MUT STAT3 were co-transfected with miR-665 mimic/mimic-NC using lipofectamine 3000 (Invitrogen, Carlsbad, CA, USA) according to the guidance of manufacturer. Luciferase activity was detected via using Dual Luciferase Reporter Assay Kit (Promega, Madison, WI, USA).

### RNA pull-down assay

The Pierce magnetic RNA protein pull-down kit was used herein for RNA pull-down assay followed by the instructions of manufacturer. The cells were lysed to collect the lysates. Subsequently, the biotinylated probes were incubated with the cell lysates and then M-280 streptavidin magnetic beads magnetic beads was added to react with the biotinylated probes complexes. After purification, the targeted RNA was detected via RT-qPCR and northern blot.

### Northern blot assay

The miR-665 was detected via northern blot assay. The separation of total RNA was processed on denaturing polyacrylamide gels (12%) and then blotted on nylon membranes (Ambion, Austin, TX, USA). After membrane hybridization was conducted in hybridization solution, labeled oligonucleotide probe was hybridized with the membranes. Gel-Pro Analyzer 4.5 software (Media Cybernetics, Rockville, MD, USA) was used for analysis of RNA quantification.

### Establishment of tumor-bearing mice

The BALB/c nude mice (6 weeks old, 20–22 g) procured from Charles River corporation (Beijing, China) were randomly arranged into control, siRNA#1, and siRNA#2 group, ten mice in each group. The right axilla of nude mice was injected subcutaneously with gastric cells (control group), gastric cells transfected with siRNA#1(siRNA#1 group) and siRNA#2 (siRNA#2 group), respectively. The density of injected cells was 3 × 10^6^. After 4 weeks, the mice were sacrificed and the weight and volume of tumor were recorded. Calipers were used to monitor the length (a) and width (b) of the tumor tissue. The formula for calculating the tumor volume is as follows: V (mm3) = 0.53*ab^2^. This study was approved by the animal ethical committee of Shandong Provincial Hospital (018-SDU-AEC-401).

### Hematoxylin-eosin staining

After the mice were sacrificed, the tumor tissues were resected and fixed in 10% formalin. After dehydration and paraffin-embedded, the samples were placed on the slicing machine to obtain 5 μm sections. Then, hematoxylin-eosin staining was conducted after dewaxing. The sections were observed under the microscope (Olympus, Tokyo, Japan).

### Immunochemical staining

The deparaffinized sections were immersed in H_2_O_2_ (3%) for 15 minutes to quench the endogenous peroxidase activity and 5% normal serum was used to occlude the nonspecific binding sites of reagents for half hour. Then, anti-Ki-67 antibody (ab238020, Abcam, Cambridge, MA, USA, 2 µg/ml) was incubated with the sections for half an hour at room temperature. After rinse, HRP-conjugated secondary antibody (ab6728, Abcam, Cambridge, MA, USA) was added to incubated with the sections. The observation of the sections was performed on the microscope (Olympus, Tokyo, Japan).

### Western blot

The cells after transfection in the study groups were lysed in radioimmunoprecipitation assay (RIPA) lysis buffer (Beyotime, Shanghai, China) for extraction of the total proteins. Bradford assay (Bio-Rad, Hercules, CA, USA) was performed for detection of protein concentrations. The proteins were isolated on 10% sodium dodecyl sulfate-polyacrylamide gel electrophoresis (SDS-PAGE) followed by transferring onto the polyvinylidene fluoride (PVDF) membranes (Millipore, Billerica, MA, USA). The membranes were occluded with 5% nonfat milk and incubated with the primary antibody against STAT3 (1:1000, ab68153, Abcam, Cambridge, MA, USA) at 4°C, overnight. Then secondary antibody (1:2000, ab6721, Abcam, Cambridge, MA, USA) was added to incubated with the membranes for 1 h. The enhanced chemiluminescence (ECL) detection system (Pierce Biotech Inc., Rockford, IL, USA) was used for visualization of bands. The density of blots was analyzed via using ImageJ analyzer software (NIH, USA).

### Statistical analysis

The analysis of the experimental results was performed via using Statistical Product and Service Solutions (SPSS) software (version 19.0) (IBM, Armonk, NY, USA) and GraphPad prism (version 8.0) (La Jolla, CA, USA). The results were presented as mean ± standard deviation (SD). Statistical significance was assessed via using one-way of variance (AVOVA) or student’s t-test. Statistically significance was denoted at p < 0.05.

## Results

### LncRNA RPSAP52 is upregulated in gastric cancer cells and deletion of lncRNA RPSAP52 suppressed cell proliferation, while accelerating apoptosis in gastric cancer cells

**Figure 1.**
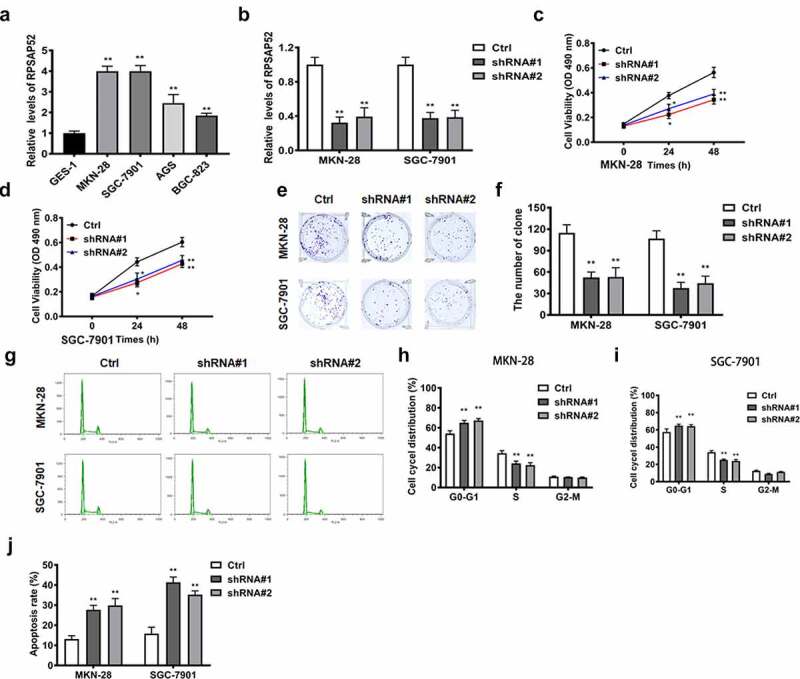
The biofunctions of LncRNA RPSAP52 in gastric cancer cells. The level of lncRNA RPSAP52 evaluated by RT-qPCR in varieties of gastric cancer cells (a). The level of lncRNA RPSAP52 evaluated by RT-qPCR following lncRNA RPSAP52 depletion (b). The cell viability at 24 and 48 h in MKN-28 (c) and SGC-7901 cells (d). The images of colony formation assay (e). The histogram for colony number (f). The cell cycle of MKN-28 and SGC-7901 cells detected by flow cytometry (g-i). The cell apoptosis assessed by flow cytometry (j). *, *p* < 0.05 and **, *p* < 0.01 vs. control group.

RT-qPCR was used to detect the expression of lncRNA RPSAP52 in GES-1, MKN-28, SGC-7901, AGS, and BGC-823 cell lines. The results showed that lncRNA RPSAP52 was significantly upregulated in four gastric cancer cell lines, among which the up-regulation fold was higher in MKN-28 and SGC-7901 cells, so these two cell lines were selected for subsequent experiments (Figure 1(a)). After shRNA#1or shRNA#2 transfection, the lncRNA RPSAP52 level was downregulated obviously in MKN-28 and SGC-7901 cells compared to control, substantiating that the transfection was successful (Figure 1(b)). The effects of lncRNA RPSAP52 knockdown on cell proliferation was examined via assessing cell viability, colony formation and cell cycle in the study groups. CCK-8 assay suggested that the cell viabilities of MKN-28 and SGC-7901 cells at 24 h and 48 h were decreased distinctively following lncRNA RPSAP52 knockdown, compared with control (Figure 1(c,d)). The colony formation assay demonstrated that the numbers of colony were reduced significantly by lncRNA RPSAP52 knockdown (Figure 1(e,f)). Meanwhile, the results of cell cycle analysis indicated that lncRNA RPSAP52 knockdown increased the cells the in the G0-G1 phase than that in S/G2-M phase (Figure 1(g,i)). Flow cytometry analysis manifested that cell apoptosis was increased significantly following lncRNA RPSAP52 knockdown (Figure (J)). These data supported that lncRNA RPSAP52 knockdown displayed inhibitory effects on cell proliferation, while accelerating apoptosis in gastric cancer cells.

### Depletion of lncRNA RPSAP52 repressed tumor growth in vivo

**Figure 2.**
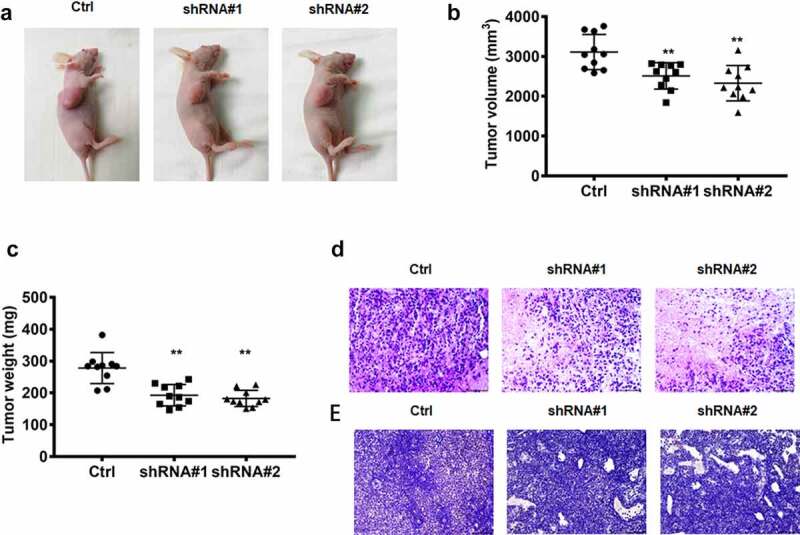
The biofunctions of lncRNA RPSAP52 in nude mice. The images of tumor-bearing mice (a); The tumor weight (b) and volume (c) in the study groups. The histopathologic changes assessed by HE staining (d); The expression level of ki-67 evaluated via immunohistochemical staining (e). **, *p* < 0.01 vs. control group.

As shown in Figure 2(a), the tumor-bearing mouse model was successfully established. The volume and weight of the tumor were reduced dramatically following lncRNA RPSAP52 knockdown compared with control (Figure 2(b,c)). Histopathological analysis demonstrated that tumor necrosis was found in the tumor tissue after lncRNA RPSAP52 knockdown, while no obvious pathological lesion change was found in tumor tissue of control group (Figure 2(d)). In addition, Ki-67, an indicator of cell proliferation capacity, was also detected herein via immunohistochemical staining. We found that Ki-67 was decreased evidently after depletion of LncRNA RPSAP52 in contrast with control (Figure 2(e)). These results indicate that lncRNA RPSAP52 knock down suppressed the growth of tumor in vivo.

### LncRNA RPSAP52 functioned as miR-665 sponge in gastric cancer cells

**Figure 3.**
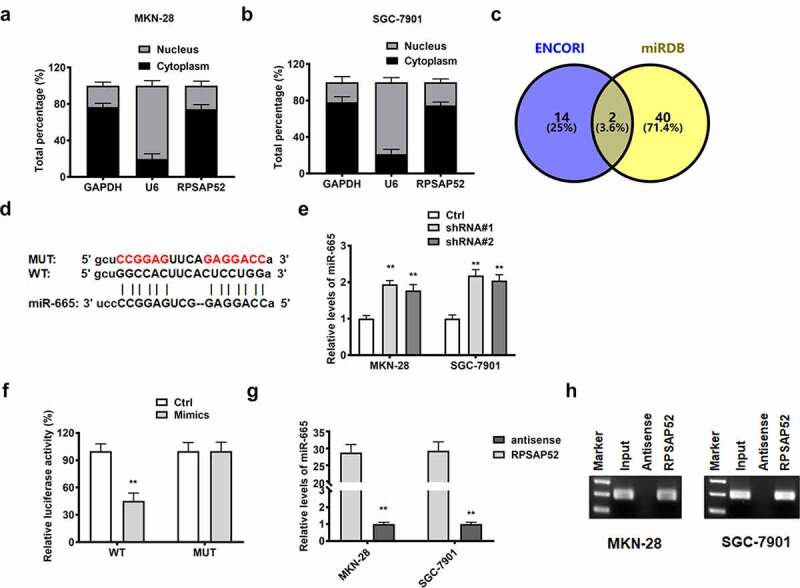
The relationship between lncRNA RPSAP52 and miR-665. The distribution percent of lncRNA RPSAP52 in cytoplasm and nucleus of MKN-28 (a) and SGC-7901 cells (b); Venn diagram of lncRNA RPSAP52 target gene in ENCORI and miRDB database (c); The binding sites between lncRNA RPSAP52 and miR-665 (d). The level of miR-665 detected by RT-qPCR (e); The luciferase report assay (f); The miRNA-665 level in complexes of RNA pull down assay was detected by RT-qPCR (g) and northern blot (h). **, *p* < 0.01 vs. control group.

In order to elucidate the functional mechanism, we performed subcellular fractionation assay and found that lncRNA RPSAP52 was high expressed in cytoplasm of MKN-28 and SGC-7901 cells (Figure 3(a,b)). Accordingly, we conjectured that lncRNA RPSAP52 may regulate gene expression at posttranscriptional level. We screened the target of lncRNA RPSAP52 via using bioinformatics tools and found that miR-665 was the potential target of lncRNA RPSAP52 (Figure 3(c,d)). The detection results of RT-qPCR showed that depletion of lncRNA RPSAP52 elevated the level of miR-665 in MKN-28 and SGC-7901 cells (Figure 3(e)), suggesting a negative association between miR-665 and lncRNA RPSAP52. We further applied luciferase reporter assay to validate the binding relationship between miR-665 and lncRNA RPSAP52. An apparent decrease of luciferase activity was found in WT-LncRNA RPSAP52 plasmid co-transfected with miR-665 mimics group compared to control group, while the luciferase activity remained unchanged in MUT-LncRNA RPSAP52 group (Figure 3(f)). RNA pull down assay was further applied to verify the binding relationship between miR-665 and lncRNA RPSAP52. As showed by results from RT-qPCR assay and northern blot, the miR-665 was enriched in lncRNA RPSAP52 group, while trace miR-665 was found in lncRNA RPSAP52 antisense group, further supporting the binding relation between miR-665 and RPSAP52 (Figure 3(g,h)). All up, miR-665 was negative regulated and targeted by lncRNA RPSAP52.

### MiR-665 mediated the effects of lncRNA RPSAP52 in gastric cancer cells

**Figure 4.**
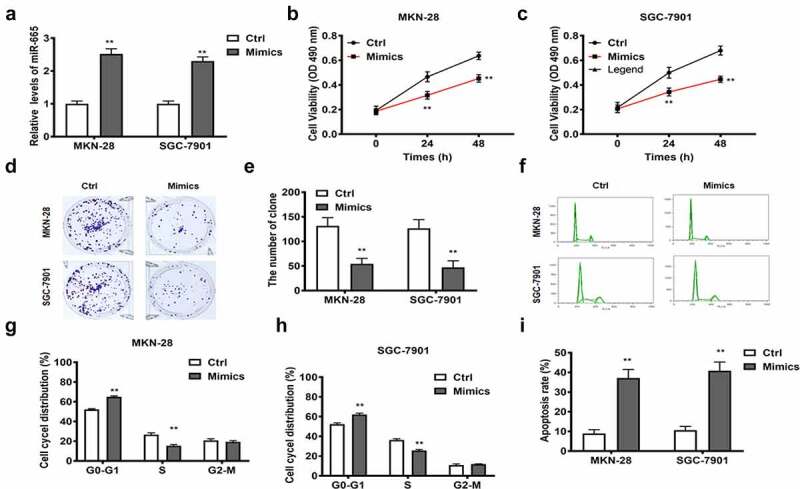
The biofunctions of miR-665 in gastric cancer cells. The level of miR-665 evaluated by RT-qPCR (a); The cell viability at 24 and 48 h in MKN-28 (b) and SGC-7901 cells (c); The images of colony formation assay (d); The histogram for colony number (e); The cell cycle of MKN-28 and SGC-7901 cells detected by flow cytometry (f-h); The cell apoptosis assessed by flow cytometry (i); **, *p* < 0.01 vs. control group.

We further explored whether the impact of lncRNA RPSAP52 in GC was mediated by miR-665. The detection results of RT-qPCR showed that the miR-665 was elevated compared to the control, suggesting the successful transfection (Figure 4(a)). The effects of miR-665 overexpression on cell proliferation and apoptosis in gastric cancer cells was further examined. CCK-8 and colony formation assays showed that the proliferation (24 h and 48 h) and colony formation ability of MKN-28 and SGC-7901 cells were inhibited after miR-665 overexpression (Figure 4(b-e)). Moreover, cell cycle detection revealed that the cells of G0-G1 phase was increased obviously following miR-665 overexpression compared to control, denoting that miR-665 induced G0-G1 arrest (Figure 4(f-h)). Flow cytometry manifested that miR-665 overexpression enhanced cell apoptosis (Figure 4(i)). Remarkably, the effects of miR-665 overexpression were in agreement with the impacts of depletion of lncRNA RPSAP52 in gastric cancer cells. We concluded that depletion of lncRNA RPSAP52 functions in gastric cancer via up-regulating miR-665.

### STAT3 acted as a target gene of miR-665

**Figure 5.**
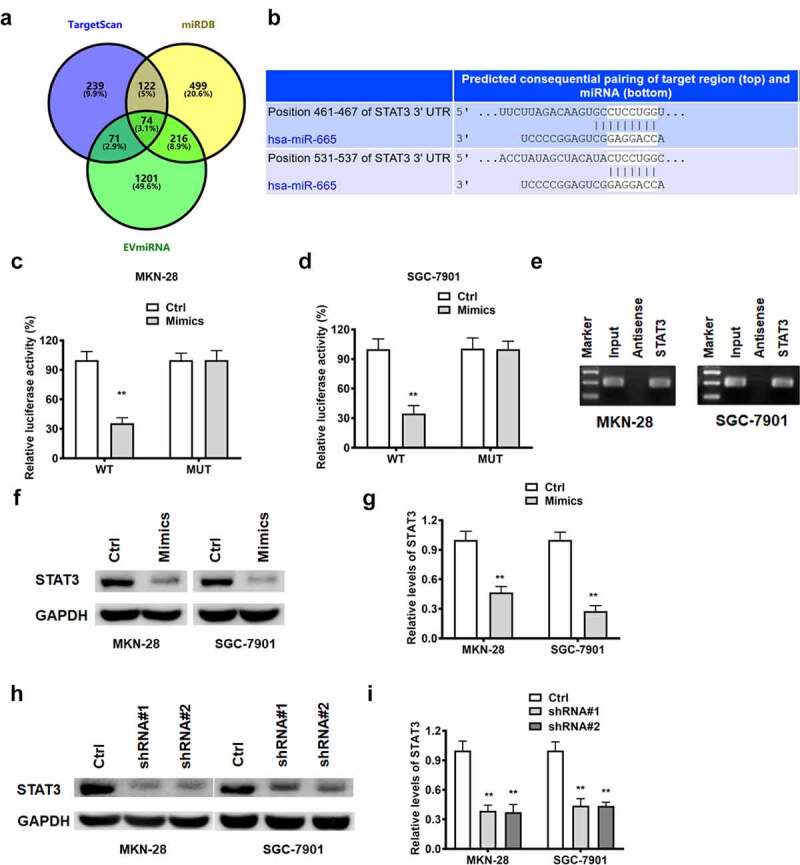
The relationship between miR-665 and STAT3.; Venn diagram of miR-665 target gene from TargetScan, miRDB and EVmiRNA database (a). The binding sites between miR-665 and STAT3 (b). The luciferase report assay in MKN-28 (c) and SGC-7901 cells (d). The miRNA-665 level in complexes of RNA pull down assay was detected by northern blot (e). The STAT3 detected by Western blot for exploring the regulatory relationship between miR-665 and STAT3 (f and g). The STAT3 detected by Western blot for exploring the regulatory relationship between lncRNA RPSAP52 and STAT3 (h and i) **, *p* < 0.01 vs. control group.

As predicted by bioinformatics online tool, STAT3 is a target of miR-665 (Figure 5(a,b)). Luciferase reporter assay was employed to validated this prediction. It was found that miR-665 mimics restrained the luciferase activity of the WT-STAT3 vector, while not of the MUT-STAT3 vector, in MKN-28 and SGC-7901 cells (Figure 5(c,d)). Norther blot results substantiated that miR-665 was enriched in STAT3 group, while none in STAT3 antisense group (Figure 5(e)). All the aforementioned results support that STAT3 is a direct target of miR-665. We further explored the regulatory relationship among lncRNA RPSAP52, miR-665 and STAT3. As demonstrated by Western blot, miR-665 overexpression and depletion of lncRNA RPSAP52 decreased the level of STAT3 in MKN-28 and SGC-7901 cells (Figure 5(f-i)), unveiling that depletion of lncRNA RPSAP52 downregulated STAT3 via upregulating miR-665.

### STAT3 functions as the downstream target of lncRNA RPSAP52/miR-665 in gastric cancer cells

**Figure 6.**
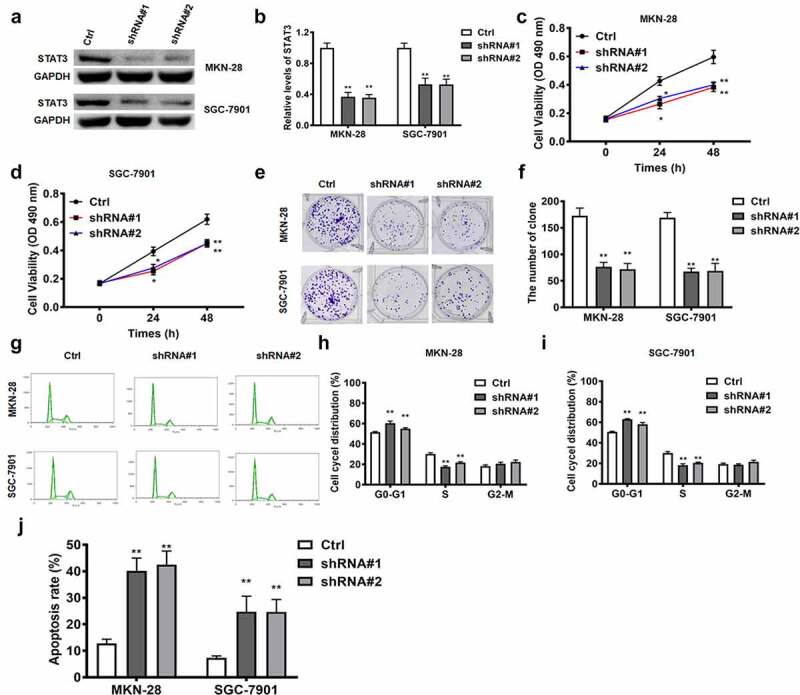
The biofunctions of STAT3 in gastric cancer cells. The level of STAT3 evaluated by Western blot (a and b). The cell viability at 24 and 48 h in MKN-28 (c) and SGC-7901 cells (d). The images of colony formation assay (e). The histogram for colony number (f). The cell cycle of MKN-28 and SGC-7901 cells detected by flow cytometry (g-i). The cell apoptosis assessed by flow cytometry (j). *, *p* < 0.05, **, *p* < 0.01 vs. control group.

LncRNA RPSAP52 depletion upregulated miR-665, which in turn downregulated STAT3. Accordingly, we further examined whether the effects of STAT3 depletion was in accordance with the effects of lncRNA RPSAP52 depletion and miR-665 mimics in gastric cancer cells. The results of Western blot showed that STAT3 decreased evidently (Figure 6(a,b)). As reveled by CCK-8 and colony formation assay, STAT3 depletion displayed inhibitory effects on the cell viability (24 h and 48 h) and colony formation capacity (Figure 6(c-f)). Besides, as presented by flow cytometric analysis results, STAT3 depletion was found to increase cell distribution at G0-G1 phase and promote cell apoptosis (Figure 6(g-j)). Taken together, the effects of STAT3 depletion on cell proliferation, apoptosis and cell cycle are consistent with LncRNA RPSAP52 depletion and mir-665 overexpression in gastric cancer cells, suggesting that STAT3 is downstream target of the LncRNA RPSAP52/mir-665 axis target gene.

## Discussion

Many studies have shown that lncRNAs play an important role in GC progression. LncRNA PROX1-AS1, highly expressed in gastric cancer, is associated with unfavorable prognosis [24]. Lnc-CTSLP4 is low expressed in tumor tissues, acting as an antioncogene in gastric cancer [25]. Long non-coding RNA KCNQ1 is reported to be upregulated in tissues and cells of gastric cancer, functioning as a contributor of gastric cancer [26]. The results of this study showed that lncRNA RPSAP52 was elevated dramatically in gastric cancer cells and lncRNA RPSAP52 depletion was found to exert anticancer effects in gastric cancer via regulating miR-665/STAT3.

LncRNA RPSAP52, a newly discovered lncRNA, has been reported to engage in several kinds of cancer including tongue squamous cell cancers, glioblastoma, and pituitary tumor [20,22,27]. While, more biological functions of LncRNA RPSAP52 remain to be researched in cancer. LncRNA RPSAP52 has been confirmed as a lncRNA antisense with respect to HMGA2 gene [21]. LncRNA RPSAP52 is found to upregulate HMGA2 via functioning as sponges of miR-16, miR-15b, and miR-15a in pituitary tumor [28]. We further assessed the level of lncRNA RPSAP52 in five types of gastric cancer cells and lncRNA RPSAP52 was found to be drastically upregulated in MKN-28 and SGC-7901 cells. Previous study demonstrates that lncRNA RPSAP52 accelerates cell proliferation via regulating cell cycle in pituitary tumor [22]. In the current study, CCK-8, colony formation and cell cycle analysis revealed that depletion of lncRNA RPSAP52 displayed proliferation promotion effect and induced G0-G1 arrest. LncRNA RPSAP52 is also involved in cell apoptosis. LncRNA RPSAP52 is reported to repress cell apoptosis in diabetic retinopathy [29]. Additionally, lncRNA RPSAP52 also exerted anti-apoptosis effects in renal proximal tubular epithelial cell under hypoxia conditions [30]. These findings manifested lncRNA RPSAP52 is a gene of apoptosis resistance. In line with this conclusion, our results demonstrated that depletion of lncRNA RPSAP52 resulted in elevation of cell apoptosis and lncRNA RPSAP52 acted as an oncogene in gastric cancer. Accordingly, we also established a model of Xenograft tumor. Our results revealed that decreased tumor weight and volume were found following lncRNA RPSAP52 depletion. LncRNA RPSAP52 depletion induced tumor necrosis and reduced the level Ki-67 which is a cell proliferation marker. Remarkably, lncRNA RPSAP52 depletion also exerted anti-cancer effects in vivo. The consistent results were found in vivo and in vitro, denoting that lncRNA RPSAP52 is a critical regulator of great clinical potential in gastric cancer.

There is mounting evidence that lncRNA commonly exerts biological effects as a competing endogenous RNA via sponging miRNA at cytoplasm level. For instance, lncRNA PAPAS is found to accelerate gastric cancer progress via sponging miRNA-188-5p [31]. LncRNA NEAT1 acts as contributor of gastric cancer via sponging miR-1224-5p [32]. LINC00858 displays oncogene effects in GC through modulating miR-363-3p [33]. As presented by results of bioinformatic prediction, lncRNA RPSAP52 was stated to target miR-665. A ceRNA-sponge mechanism of lncRNA RPSAP52 on miR-665 has been validated via RNA pull down and luciferase report assay.

MiRNA-665 has been substantiated as a crucial player in sundry cancers such as hepatocellular cancer, ovarian cancer, colorectal cancer [^34–36^]. MiR-665 has been demonstrated to exert anti-proliferation effect in gastric cancer via targeting PPP2R2A [37]. This corresponds to our results that miR-665 suppressed cell proliferation and colony formation capacity, and induced G0-G1 arrest. MiR-665 is also involved in cell apoptosis. It is found that LINC00565 regulates cell apoptosis via targeting miR-665 [38]. Consistent with our findings, miR-665 overexpression enhanced cell apoptosis drastically. Obviously, lncRNA RPSAP52 depletion and miR-665 overexpression function identically in gastric cancer, supporting that lncRNA RPSAP52 depletion functions via elevation of miR-665.

STAT3 has been confirmed as a well-known oncogene factor. STAT3 is reported to aggravate progress of gastric cancer via promotion of mesothelial–mesenchymal transition [39]. STAT3 is found to expedite metastasis and cell proliferation in gastric cancer [40]. STAT3 depletion is confirmed to enhance cell apoptosis in gastric cancer [41]. Conformance to these previous findings, in this report, STAT3 depletion reduced cell proliferation, weakened colony formation capacity, induced G0-G1 phase arrest and promoted cell apoptosis. As denoted by the results of bioinformatics analysis, there is a binding relationship between miR-665 and STAT3. STAT3 was further verified as the direct target via luciferase report assay and RNA pull down assay. We further found that STAT3 was negatively regulated by miR-665 which was further sequestered by lncRNA RPSAP52. Remarkably, STAT3 depletion, lncRNA RPSAP52 depletion and miR-665 overexpression functions equally in gastric cancer. Thus, it is concluded that lncRNA RPSAP52 depletion exerted biological effects via regulating miR-665/STAT3.

## Conclusions

In this study, we identified a new regulatory axis lncRNA RPSAP52/miR-665/STAT3 in gastric cancer. lncRNA RPSAP52 exerted anti-cancer effects via regulating miR-665/STAT3 in GC. This discovery provides a new idea for the study of the anticancer mechanism of GC, and lays a foundation for the study of the pathological mechanism of GC.

## Highlights

• LncRNA RPSAP52 is upregulated in gastric cancer cells, and deletion of LncRNA RPSAP52 inhibits cell proliferation and promotes cell apoptosis;
• Depletion of lncRNA RPSAP52 repressed tumor growth in vivo;
• LncRNA RPSAP52 functioned as miR-665 sponge in GC cells;
• MiR-665 mediated the effects of lncRNA RPSAP52 in GC cells;
• STAT3 functions as the downstream target of lncRNA RPSAP52/miR-665 in GC.
